# *Bruchus rufimanus* Boh. Effect on Broad Bean Seed Quality and the Infection Level of Seed-Borne Fungal Pathogens

**DOI:** 10.3390/plants12091825

**Published:** 2023-04-29

**Authors:** Mohammad Almogdad, Akvilė Jonavičienė, Roma Semaškienė

**Affiliations:** Department of Plant Pathology and Protection, Institute of Agriculture, Lithuanian Research Centre for Agriculture and Forestry, Akademija, LT-58344 Kėdainiai District, Lithuania; akvile.jonaviciene@lammc.lt (A.J.); roma.semaskiene@lammc.lt (R.S.)

**Keywords:** broad bean weevil, damage, fungi, germination, *Vicia faba*, vigor

## Abstract

Broad bean weevil (*Bruchus rufimanus* Boh.) is considered an economically important insect pest of the broad bean crop. Its damage starts when larvae burrow into the seed and feed on endosperm or kill the embryo, thus the seed cannot germinate. The microbiological quality and consumer safety of broad bean may be compromised by holes. This study was performed during 2018–2020 to estimate the effect of seed damage by *B. rufimanus* on seed quality, germination rate and seedling growth, and on the incidence of seed-borne fungal pathogens. Broad bean seeds were collected and classified as damaged by *B. rufimanus* or non-damaged. There was a relationship between broad bean weevil damage and an increase in fungal contamination. Results showed that germination decreased from 97.2% for non-damaged seeds to 81.4% for the seeds with *B. rufimanus* damage. Seven fungal genera were identified: *Fusarium* spp., *Alternaria* spp., *Aspergillus* spp., *Penicillium* spp., *Cladosporium* spp., *Mucor* spp. and *Botrytis* spp. The most common fungal genus was *Cladosporium* spp. Our research shows that *B. rufimanus* negatively affects the germination of its host’s seeds and decreases the development of seedlings.

## 1. Introduction

The majority of crops are grown from seed, and the primary basic materials for both human and animal feed are cereal and legume grains [1]. Broad bean (*Vicia faba* Linnaeus) is a leguminous plant that blooms once a year. It is one of the oldest crops worldwide [2] and is well known, especially in Europe, Asia, and North Africa [3]. Broad bean is produced in Europe mainly in moist and moderate conditions [4]. Globally, in 2017, broad bean was grown on 2.4 million ha [5]. The yield of broad bean in Lithuania was about 3.75 t ha^−1^ from 58.3 thousand ha of land [6]. Broad bean is a valuable food crop that provides positive value for human and animal diets because of its nutritious seeds, which have a high protein and carbohydrate content [7] and are a good source of vitamins and minerals [8]. Broad bean is cultivated mainly for its mature seeds, which are consumed as a snack, or for its green pods [9]. Broad bean is also used as green manure [10] because of the symbiotic nitrogen-fixing bacteria present in the root nodules, which can convert 200 million tons of nitrogen per year into ammonia [11]. So, it is important crop in crop rotation, which helps to minimize the need for fertilizers [12].

More than 70 species of insect pest occur in broad bean, causing damage at all growth stages [13]. Broad bean weevil (*Bruchus rufimanus* Boh.) is associated with broad bean and is distributed worldwide, including in Europe. It significantly decreases the yield and is considered economically important insect pest [14]. Adults of *B. rufimanus* spread to broad bean fields at the beginning of the flowering stage [15]. Females lay eggs directly onto the exterior side of pods [16]. Larvae burrow into the seed, feeding on endosperm or killing the embryo, thus the seed cannot germinate [17], resulting in a 45% to 70% lower yield compared to non-damaged seeds [18]. The percentage of damaged seeds ranges from 18.5% to 28.9% [19]. In addition, the damaged seeds have a 13% lower germination rate compared to non-damaged seeds [18]. As larvae complete their developmental cycle within the seed, they cause losses of mass and reduce the fodder value of the seeds [19]. Germination rate losses depend on the size of seed and portion remaining after larval feeding [16]. In addition, seeds damaged by *B. rufimanus* will be more likely to be infected by pathogens [19]. They are more susceptible to fungal pathogens, such as moulds [20], and other phytopathogenic organisms [18]. The presence of *B. rufimanus* in the seeds reduces the commercial value of broad bean yield [21].

Several biotic factors lead to low productivity: fungal diseases play the main role, as along with insect pests, by preventing germination or by having a negative impact on the strength of seedling growth or reducing the storability [22,23,24]. A large variety of microorganisms, particularly fungi, may exist inside seeds. The spread of plant diseases, the degradation of seed quality, and the shortening of seed life are the three primary negative impacts of seed-associated fungi [1]. The common fungal genera isolated from broad bean seeds are *Aspergillus* spp., *Epicoccum* spp., *Fusarium* spp., *Alternaria* spp., *Trichoderma* spp., *Penicillium* spp., *Botrytis* spp., *Cephalosporium* spp., *Cladosporium* spp., *Rhizoctonia* spp., *Stemphylium* spp., *Trichothecium* spp., *Ascochyta* spp., *Phoma* spp. and *Colletotrichum* spp. [25,26,27]. Nine seed-borne diseases, caused by 13 fungal pathogens, that can infect broad bean have been recorded [26]. Anthracnose, chocolate spot, root rot and leaf rot are considered the major diseases. The entire yield can be lost when planting seeds containing the anthracnose pathogen under appropriate conditions for its development [28]. *Fusarium* spp., cause damping-off of the seedlings and a decrease in germination energy [29]. All of these fungi are seed-transmitted and can exist as mycorrhizal fungi on the seed’s surface or as conidiophores in the sheath [30]. The production of broad bean is restricted by all of these fungi in many countries [31].

The use of disease-free seeds and a very good germination rate are considered the main factors for increasing crop productivity and profitability. This study was performed to estimate the effect of seed damage by *B. rufimanus* on seed quality, germination rate and seedling growth, and on the incidence of seed-borne fungal pathogens, to improve our understanding and to prevent some fungal diseases in broad bean.

## 2. Results

### 2.1. Weather Data

**Figure 1:**
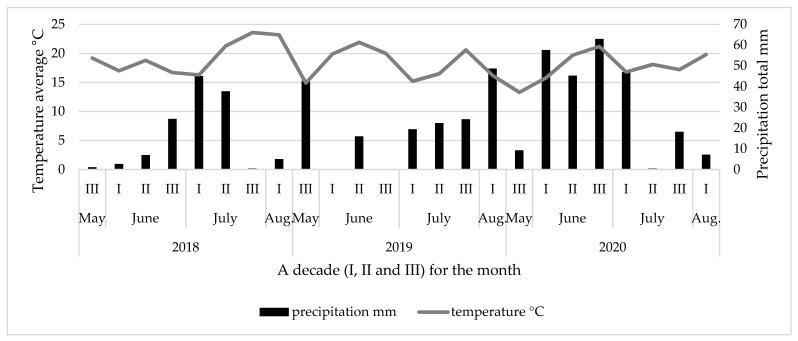
Temperature and precipitation during the growing season (May–August) in 2018–2020.

The average air temperature and the total precipitation during the 2018–2020 growing seasons are given in Figure 1.

In 2018, warm, dry, sunny, and windy weather was prevalent in May, with temperatures averaging 4.9 °C higher than normal and only 1.1 mm of rainfall. June was also warm, dry, and sunny, with temperatures averaging 1.6 to 3.5 °C higher than normal and minimal rainfall. The last third of June had changeable weather, but the dry period lasted until the end of the month. July had a mix of warm and rainy weather, with some days receiving significantly more precipitation than normal. August was hot and windy, with below-normal rainfall. Overall, there was a total of 119.8 mm of precipitation and an average air temperature of 19.7 °C from flowering to harvesting. In 2019, June had hot and dry weather, with temperatures averaging, compared to the same month in the three previous decades, 4.5, 6.6 and 3.7 °C, respectively, higher than normal, with minimal rainfall. July had changeable weather, with the first ten days being rainy and the second ten days being warm and dry. The last third of July had below-normal rainfall. August was dry and warm, with below-normal rainfall. Overall, there was 133.7 mm of rainfall and an average air temperature of 18.6 °C from flowering to harvest time. In the 2020 season, June had a mix of warm and rainy weather, with the second third being hot and rainy and the last third being hot and dry, except for the last day, which had significant rainfall. July had changeable weather, with the first third being rainy and the second third being warm and dry. The last third of July had below-normal rainfall. August was dry and warm, with below-normal rainfall. Overall, there was 229.6 mm of rainfall and an average air temperature of 18.6 °C from flowering to harvest time.

### 2.2. Seeds Germination and Seedling Vigor

The impact of damage by *B. rufimanus* on the weight of broad bean seeds and their germination was recorded (Table 1). The results showed significant differences in germination rate between damaged and non-damaged seeds for all study years. The germination rate ranged from 94.5 to 99.0% for non-damaged seeds whereas, for damaged seeds, the rate was 69.0 to 89.5%. In 2018, 2019, and 2020, the germination rate of damaged seeds was observed to be reduced by 9.6%, 27.0%, and 12.7%, respectively, as compared to non-damaged seeds. Our study results showed that the highest average germination rate was (97.2%) in the non-damaged seeds whereas a significant seed germination rate reduction was recorded in damaged seeds. The combined data showed that the germination rate was strongly correlated with the damage to broad bean seeds by *B. rufimanus* for all study years (r = 0.70, *p* value ≤ 0.01, *n* = 48). In 2020, the germination index of damaged broad bean seeds was only slightly lower (12.4) than that of non-damaged seeds (12.8). However, in 2018 and 2019, the damage had a greater impact, causing the germination index to decrease to less than 85%, with values of 12.3 and 9.2, respectively, in damaged seeds. The thousand-grain weight (TGW) of seeds was significantly influenced by *B. rufimanus* damage in all study years. The damaged seeds exhibited a significantly lower TGW, with an average decrease of 19.7%, to 507.75 g, compared to non-damaged seeds that had a TGW of 631.66 g.

Biometric results for shoot and root express the strength of the seedlings from non-damaged and damaged seeds by *B. rufimanus* (Table 2). Regarding the length of shoot and root, the highest values were recorded in 2018 (26.1 and 17.2 cm respectively). Concerning the root length, significant differences were recorded only in 2018. In 2019 and 2020, significantly higher shoot lengths were established for non-damaged seeds compared to damaged seeds. Concerning shoot weight, the highest weight was recorded from non-damaged seeds in 2020 (20.8 g), with significant differences compared to damaged seeds (15.4 g). In 2019 and 2020, significantly higher root weights were obtained from non-damaged seeds compared to damaged seeds. Non-damaged seeds had significantly higher values for the vigour index of shoot and root than damaged seeds in all study years except for root vigour in 2020. The shoot vigour indexes ranged from 1265.2 (damaged seeds) to 2581.4 (non-damaged seeds), whereas the root vigour indexes ranged from 752.1 (damaged seeds) to 1699.5 (non-damaged seeds).

### 2.3. Fungal Contamination on Seed

The data suggest that seeds damaged by *B. rufimanus* showed a tendency to be more infected by pathogens than non-damaged seeds (Table 3). Seven fungal genera were identified from the seeds, *Fusarium* spp., *Alternaria* spp., *Aspergillus* spp., *Penicillium* spp., *Cladosporium* spp., *Mucor* spp. and *Botrytis* spp., out of these *Cladosporium* spp. was predominant. From the *Fusarium* species complex, *F. avenaceum*, *F. equiseti*, *F. poae*, *F. oxysporum*, *F. sporotrichioides* and *F. graminearum*. *F. equiseti* and *F. avenaceum* were regarded as dominant species. In our study, seed infection with fungal pathogens was closely related to meteorological conditions. *Fusarium* infection on seeds was higher in 2020 than in all other investigation years. In 2020 (Figure 1), as opposed to 2018 and 2019, there was a higher amount of rainfall. Warm and wet weather at flowering time creates favourable conditions for this pathogen to spread in broad bean fields. A similar tendency was visible with seed infection by *Botrytis* spp. *Penicillium* spp. infected more seeds in 2019, when rainy weather prevailed at harvest time. On the contrary, *Cladosporium* spp. was found in higher amounts in 2018 and 2020, when the amount of rainfall near harvest time was less than in 2019. Analysis of non-damaged seeds and seeds damaged by *B. rufimanus* showed, that in 2018, for the surface sterilized seeds, the damaged seeds had a significantly higher infection by *Alternaria* spp., *Penicillium* spp., *Cladosporium* spp. and *Botrytis* spp. compared to the non-damaged seeds. For the non-surface sterilized seeds, the non-damaged seeds had a significantly lower infection by *Penicillium* spp. and *Cladosporium* spp. than the damaged seeds. For the other fungal pathogens, no significant differences were found between the damaged and non-damaged seeds. In 2019, the results showed that *Fusarium* spp., *Alternaria* spp. and *Botrytis* spp. were present in significantly higher amounts in the surface-sterilized seeds that had been damaged by *B. rufimanus* compared to the non-damaged seeds. The non-surface-sterilized broad bean seeds that had been damaged by *B. rufimanus* had significantly a higher incidence of *Alternaria* spp. and *Aspergillus* spp. compared to the non-damaged seeds. In 2020, the most frequently identified fungal genus was *Cladosporium* spp. For the surface sterilized and non-sterilized seeds, the damaged seeds had higher infection by *Fusarium* spp. and *Alternaria* spp. with significant differences compared to non-damaged seeds.

## 3. Discussion

Seed germination is affected by abiotic factors and damage by insect pests as part of the biotic factors [32]. Our results showed that the seeds damaged by *B. rufimanus* had lower germination rate compared to the non-damaged seeds. Similar results were reported by Khelfane-Goucem and Medjdoub-Bensaad [17], who found that the germination rate of broad bean seeds was reduced by *B. rufimanus* larvae damage. Other previous studies have shown the negative, 55–90.5%, effects of *B. rufimanus* on seed germination rate [33]. Titouhi et al. [34] reported that seeds damaged by *B. rufimanus* had germination rates ranging from 32.6 to 26.76%. The seeds damaged by *B. rufimanus* had significantly lower germination rates compared to non-damaged seeds, independently of the variety. Considering the outcomes of the germination test shown above, the decrease in seed germination with *B. rufimanus* was similar to the results of Titouhi et al. [34]. According to their findings, the damage significantly inhibited seed germination. The germination reduction ranged between 10.2 and 32.98%. The germination rate reduction caused by bruchids may be impossible to predict [35]. Bruchid larvae feed on a large part of the endosperm and may kill the embryo of seed, which then cannot germinate [36]. The failure of seed germination may be due to microorganisms entering from the holes created by *B. rufimanus*, similarly to what happens when wheat seeds are attacked by weevils [37]. Similar results were found by Jha et al. [38], who reported that reductions in wheat cultivar germination were brought on by rice weevil infection. In another legume crop, pea (*Pisum sativum* L.), different varieties damaged by pea weevil (*B. pisorum*) had poor germination [39]. Similar results were found by Mateus et al. [40], who reported that non-damaged pea seeds had a higher germination rate compared to seeds damaged by *B. pisorum*. Regarding the seeds’ weight, our results showed that *B. rufimanus* caused severe damage to broad bean seeds, which was illustrated by weight loss. The results of our research agreed with previous studies. Titouhi et al. [34] reported that the weight reduction in seeds damaged by *B. rufimanus* reached 9.67%. Similar results were found by Ward [14], who showed that a reduction in thousand-grain weight was found in the seeds damaged by *B. rufimanus* compared to non-damaged seeds. As a result of endosperm consumption by the feeding larvae, the weight of the seeds is decreased [41]. Suppression of shoot and root growth, as well as a reduction in their weight, resulting from *B. rufimanus* damage to grain, was not clear. The length and weight of the shoots and roots of damaged seeds was slightly lower than in non-damaged seeds. Consumption of the seed’s nutrients in damaged seeds may slow the process of starting the development of plant [32]. The low negative effect of *B. rufimanus* attack on the length and weight of shoot and root may be due to the cotyledon’s ability to mitigate the shock of an attack [42]. In support of these results, the vigour index of shoot and root was significantly higher for non-damaged seeds than for the damaged seeds. Similar results were found by Nikolova [39], who reported that pea plants that were grown from seeds damaged by *B. pisorum* had lower vigour than non-damaged seeds. Regardless of varieties, the damage by *B. rufimanus* was connected to the reductions in shoot and root vigour and the likelihood of germination.

The fungi isolated from broad bean seeds included *Fusarium* spp., *Alternaria* spp., *Aspergillus* spp., *Penicillium* spp., *Cladosporium* spp. and *Mucor* spp. These results corresponded with other studies [43,44].

In all study years, the non-damaged seeds (no *B. rufimanus* damage) revealed a lower frequency of fungal contamination. For non-sterilized broad bean seeds, in 2018, the damaged seeds revealed a significant frequency of fungal contamination (*Penicillium* spp. and *Cladosporium* spp.). In 2019, the non-damaged seeds revealed a significantly lower frequency of fungal contamination (*Alternaria* spp. and *Aspergillus* spp.). The same trend was found in 2020; the seeds damaged by *B. rufimanus* presented a higher fungal contamination rate compared to the non-damaged seeds. For sterilized broad bean seeds with the presence of *B. rufimanus* damage, the contamination by fungal pathogens increased in all study years. The same behaviour was seen for coffee beans [45]. *Cladosporium* spp. exhibited the opposite behaviour in non-sterilized broad bean seeds in 2019, but on a lower scale. The most significant agroecological element affecting the life cycle phases of fungi and their capacity to invade crops and thrive is the climate (temperature, accessible water and light intensity, as well as humid/dry cycles). It is possible that the spread of pathogenic fungi and the pattern of mycotoxin incidence will vary as a result of their capacity to adapt to climate change [46]. Numerous variables (broad bean sensitivity, insect pest damage, drying, storage, etc.) and the environment affect the pathogens found in broad bean [45]. Since the research location has a unique annual average temperature, rainfall, and humidity, the larger proportion of contamination in non-sterilized broad bean seeds may be related to the climate. However, cultural activities, such as appropriate agricultural methods used before and after harvest, also influence fungal infection [47]. Previous research has examined the relationships between meteorological variables, broad bean seed production and the presence of saprophytic and pathogenic fungi. Pszczółkowska et al. [48] reported that rainfall and temperature have an impact on the occurrence of fungi, and other pathogens, that produce toxins, and saprophytes. Seven distinct fungal genera were found in broad bean seeds that were contaminated internally or externally. The majority genera of the isolates of fungi were *Cladosporium* spp. and *Penicillium* spp., which were well spread through non-sterilized seeds (non-damaged seeds or seeds damaged by *B. rufimanus*). Additionally, these genera were identified from sterilized seeds (damaged or not). They have been identified as the most common fungus in several other studies [25,49]. *Alternaria* spp. was a third fungus that was prevalent and isolated in all of the tested seeds [50]. This genus is frequently connected to broad bean seeds and has the potential to infect broad bean plants with a leaf spot disease. Honda et al. [51], when isolating fungi associated with broad bean based on its cultural and morphological characteristics, reported that *Alternaria tenuissima* was isolated in all 15 fields surveyed. Since the damaged seeds show a higher fungal infection rate, in either the sterilized or non-sterilized seeds, compared to the non-damaged, the relationship between *B. rufimanus* damage and the rise in the percentage of fungal diseases was noticeable. Our results are in line with those of Pérez et al. [52], who found that coffee berry borer (*Hypothenemus hampei*) promoted the infection by fungi in coffee seeds. Previous reports noted an increase in the number of *Fusarium* spp. infections as a result of greater bruchid damage [53]. The primary finding of the current investigation is the loss of distinctive seed quality due to *B. rufimanus* damage and its association with the associated fungal infections. Plant diseases and insect herbivores can interact directly or indirectly [54]. Herbivores and plant diseases can change the biochemistry of plants. The incidence, adaptability, and function of the microbes are therefore impacted by these modifications, which have an impact on the induction of defensive signaling pathways in the plant [55].

## 4. Materials and Methods

### 4.1. Field Experiment

A broad bean field was laid out by design with four replicates of 50 m^2^ plots in the experimental field of Institute of Agriculture, Lithuanian Research Center for Agriculture and Forestry during in Akademja, Kėdainiai, Lithuania during 2018–2020. The region is 63 m above mean sea level and is located at latitude 55° north and longitude 23° east. The study was carried out on the broad bean variety ‘Tiffany’. The sowing rate was 0.5 million seeds per hectare and the required agronomic measures were applied. Plants satisfied their water requirement from rainfall without additional irrigation. The sowing dates were 24 April in 2018 and 2019 and 9 April in 2020. The soil and climatic conditions (loam soil with 4.03% of organic matter, pH 6.8, K_2_O 190 mg kg^−1^ and P_2_O_5_ 192 mg kg^−1^) were the same. Fertilizer, herbicides, and fungicides were applied, following the recommended agronomic practices, but insecticides were not used during the growing season. The harvest time was similar in all research years; bean grains were harvested on 10–13 of August. After harvesting each year, broad bean seeds were divided into two groups: seeds damaged by a *B. rufimanus* emergence hole (group 1) and non-damaged seeds (group 2). The laboratory tests on the seeds were performed 5 months after harvesting. Average mean daily temperatures for the months (May, June, July, and August) of the broad bean growth season for each year were obtained from an official meteorological station located about 0.5 km away from the experimental site.

### 4.2. Seed Quality

To prevent the effect of dormancy on germination, the seeds were incubated at 10 °C for 3 days before the test. For each replication of the two groups, seeds damaged by *B. rufimanus* and non-damaged seeds, the germination rate was estimated by taking 400 seeds (50 seeds in each replicate). The seeds were put in trays on sterile sand then incubated for 8 days at a temperature of 20 °C according to the ISTA rules [56]. Growing media was checked regularly to ensure there was sufficient moisture for germination. The germinated seeds were counted 4 and 14 days after sowing [56]. A seed was determined to have germinated after the radicle measured 10 mm in length [32]. The germination rate (GR) was estimated using Formula (1) [57]:

(1) $$GR=\left[\frac{n}{N}\right]\times 100$$

where *n*—is the number of germinated seeds and *N*—is the total number of seeds.

For each group of seeds, the germination index (GI) was calculated according to the Association of Official Seed Analysts as described by Javaid et al. [58] using Formula (2):

(2) $$\mathrm{GI}=\frac{\mathrm{Number}\;\mathrm{of}\;\mathrm{germinated}\;\mathrm{seeds}}{\mathrm{Days}\;\mathrm{of}\;\mathrm{the}\;\mathrm{first}\;\mathrm{count}}+\cdots \cdots \cdots +\frac{\mathrm{Number}\;\mathrm{of}\;\mathrm{germinated}\;\mathrm{seeds}}{\mathrm{Days}\;\mathrm{of}\;\mathrm{the}\;\mathrm{final}\;\mathrm{count}}$$

Length of plumule and radicle was recorded. The root length was measured from the root cap to the end of white hypocotyl. Weight of roots and shoots was measured. Formula (3) was used to determine the main radicle and plumule vigour indexes (VI) [59]:

(3) VI=GR×L

where *GR*—is the germination rate (%) and *L*—is the length of primary radicle or plumule (cm).

With regard to thousand grain weight, four replicate samples from non-damaged seeds and another four samples from seeds damaged by *B. rufimanus* were collected randomly from the whole harvest and put through the Contador machine to count 1000 seeds from each sample. Then, the weight of 1000 seeds from each sample was recorded to the same number of decimal places (0.01 g). For weight accuracy, the sample was divided into two parts, with 500 seeds in each part, and weighed separately. If the difference between the two weights for the same sample was more than 5.00 g, the counting and weighing were repeated.

### 4.3. Seed-Borne Pathogen Identification

A total of 1600 seeds were collected randomly from the whole harvest in 2018, 4800 seeds in 2019 and 3200 seeds in 2020. In each season, the collected seeds were divided into two equal groups, seeds damaged by *B. rufimanus* and non-damaged seeds. Each one of these groups was also divided into two equal subgroups, sterilized and non-sterilized. Dividing the seeds into these subgroups allowed us to properly test our hypothesis that damage by *B. rufimanus* beetles would increase susceptibility to fungal pathogens, even with seed sterilization. We aimed for equal sample sizes in each subgroup to maintain sufficient statistical power for these comparisons. To determine the seed-borne fungi, tests were carried out according to the International Seed Testing Association (ISTA) [60,61] using the agar plate method. For identification of the fungi, the colony characters and morphology of sporulating structures were examined under a microscope (Olympus SZ61 and Nikon Eclipse E200) using classification keys [61,62]. For sterilizing the surface, seeds were treated with 1% sodium hypochlorite solution for 3 min followed by 3 successive rinses with sterilized water. Then, they were dried between the layers of sterile blotting paper. The seeds in each subgroup were plated in Petri dishes (9 cm diameter) with potato dextrose gar (PDA) [63]. Five seeds per dish were plated. The dishes were incubated at 22 ± 1 °C with 12 h cool white fluorescent light and 12 h darkness, periodically, for 7 days. The percentage of broad bean seeds infected by each fungal genus in each subgroup was recorded.

### 4.4. Statistical Analysis

SAS statistical software 9.4 was used to record and statistically analyze the data (SAS Institute Inc., Cary, NC, USA). Prior to analysis, the data were examined for homogeneity using the Kolmogorov–Smirnov Test. Duncan’s multiple range test was used to determine the significance of differences at alpha ≤ 0.01. Correlation analyzes between germination rate and *B. rufimanus* damage were calculated in Microsoft Excel 365.

## 5. Conclusions

Seeds damaged by *B. rufimanus* negatively affected broad bean germination and seedling growth. The results of the study showed that broad bean weevils influenced broad bean seed weights negatively, to varying degrees. It can be suggested that damage due to *B. rufimanus* is harmful to broad bean seed germination. There is a strong inverse relationship between *B. rufimanus* damage and broad bean seed germination. The seed damage done by *B. rufimanus* significantly decreased the germination rate by up to 27% (from 94.5% to 69%), the length and weight of shoot by 24.8% and 24.1% and the vigour index of shoot and root by 44.7% and 35.3%, respectively, in each subgroup. Seeds damaged by *B. rufimanus* had lower sowing characteristics (low germination rate and low vigour indexes). In our research, the infection level of pathogens was influenced by *B. rufimanus* seed damage. The holes made by *B. rufimanus* adults made it easier for microorganisms to enter to the tissues, so the fungal infections in the tissues of those grains increased. During the years of our research, *Cladosporium* spp., *Penicillium* spp. and *Alternaria* spp. occurred more abundantly on broad bean seeds than other fungal species.

## Figures and Tables

**Table 1:** Impact by *Bruchus rufimanus*-damaged seeds on quality of broad bean, 2018–2020.

Year	Germination Rate (%)		Germination Index		Thousand Grain Weight (g)	
	Non-Damaged Seeds	Damaged Seeds	Non-Damaged Seeds	Damaged Seeds	Non-Damaged Seeds	Damaged Seeds
2018	99.0 ± 0.37 a	89.5 ± 1.67 b	13.2 ± 0.13 a	12.3 ± 0.60 b	647.9 ± 16.53 a	539.5 ± 9.69 b
2019	94.5 ± 1.05 a	69.0 ± 3.72 b	12.5 ± 0.39 a	9.2 ± 0.67 b	609.3 ± 8.63 a	472.8 ± 2.39 b
2020	98.2 ± 2.20 a	85.7 ± 3.91 b	12.8 ± 0.47 a	12.4 ± 0.60 a	637.7 ± 7.06 a	511.0 ± 4.85 b
Average	97.2	81.4	12.8	11.3	631.7	507.7

Note. Means within each row followed by the same letter(s) are not significantly different at *p* ≤ 0.01; ± standard deviation.

**Table 2:** Impact of *Bruchus rufimanus*-damaged seeds on the length, weight and vigor index of shoot and root of broad bean, 2018–2020.

Year	Length (cm)		Weight (g)		Vigor Index	
	Non-Damaged Seeds	Damaged Seeds	Non-Damaged Seeds	Damaged Seeds	Non-Damaged Seeds	Damaged Seeds
**Shoots**						
2018	26.1 ± 0.5 a	25.5 ± 0.7 a	17.8 ± 0.7 a	15.5 ± 0.8 a	2581.4 ± 57.6 a	2276.8 ± 49.2 b
2019	24.2 ± 0.7 a	18.2 ± 1.4 b	20.3 ± 0.5 a	15.4 ± 1.3 b	2289.7 ± 79.4 a	1265.2 ± 121.2 b
2020	23.4 ± 1.2 a	17.9 ± 1.0 b	17.3 ± 1.4 a	11.6 ± 0.9 b	2260.5 ± 137.7 a	1676.1 ± 112.1 b
Average	24.6	20.53	18.5	14.2	2377.2	1739.4
**Roots**						
2018	17.2 ± 0.4 a	13.6 ± 0.4 b	5.0 ± 0.6 a	4.4 ± 0.4 a	1699.5 ± 39.3 a	1222.6 ± 49 b
2019	11.9 ± 0.7 a	10.5 ± 0.5 a	12.3 ± 0.3 a	8.8 ± 0.6 b	1114.6 ± 64.4 a	752.1 ± 47.5 b
2020	17.9 ± 0.6 a	17.1 ± 0.6 a	11.2 ± 0.6 a	5.6 ± 0.3 b	1680.2 ± 66.2 a	1543.1 ± 65.9 a
Average	15.7	13.7	9.5	6.3	1498.1	1172.6

Note. Means within each row followed by the same letter(s) are not significantly different at *p* ≤ 0.01; ± standard deviation.

**Table 3:** Percentage (%) of broad bean seeds infected by fungal pathogens, categorized by *Bruchus rufimanus*-damage status, in 2018–2020 and described by mean values.

Year	Fungal Pathogen	Non-Sterilized Seeds		Sterilized Seeds	
		Damaged Seeds	Non-Damaged Seeds	Damaged Seeds	Non-Damaged Seeds
2018	*Fusarium* spp.	5.8 ± 4.3 CD a	1.5 ± 0.6 C ab	3.0 ± 2.4 D ab	0.0 ± 0.0 B b
*n* = 400 *	*Alternaria* spp.	37.3 ± 5.9 B a	27.0 ± 8.6 B a	24.3 ± 9.8 B a	1.8 ± 1.0 B b
	*Aspergillus* spp.	6.0 ± 1.4 CD a	7.0 ± 0.8 C a	0.3 ± 0.5 D b	0.0 ± 0.0 B b
	*Penicillium* spp.	44.0 ± 5.4 B a	30.0 ± 0.8 B b	13.3 ± 4.0 C c	0.5 ± 0.6 B d
	*Cladosporium* spp.	96.3 ± 2.6 A a	64.5 ± 7.5 A b	53.3 ± 7.2 A b	24.0 ± 2.2 A c
	*Mucor* spp.	14.3 ± 13.7 C a	9.3 ± 6.8 C a	0.0 ± 0.0 D a	0.3 ± 0.5 B a
	*Botrytis* spp.	0.5 ± 1.0 D ab	0.0 ± 0.0 C b	1.8 ± 1.0 D a	0.0 ± 0.0 B b
2019	*Fusarium* spp.	1.3 ± 1.7 C ab	0.3 ± 0.7 C b	1.8 ± 1.7 C a	0.3 ± 0.5 D b
*n* = 1200 *	*Alternaria* spp.	50.7 ± 17.2 B a	19.6 ± 13.4 C b	59.1 ± 12.8 A a	11.0 ± 9.3 A b
	*Aspergillus* spp.	3.3 ± 2.1 C a	0.6 ± 0.9 C b	0.3 ± 0.7 C b	0.9 ± 1.1 CD b
	*Penicillium* spp.	88.3 ± 10.1 A a	75.9 ± 25.9 A a	23.3 ± 18.2 B b	5.8 ± 5.1 BC b
	*Cladosporium* spp.	39.2 ± 22.1 B a	48.2 ± 33.5 B a	14.3 ± 5.7 B b	8.4 ± 3.8 AB b
	*Mucor* spp.	8.2 ± 10.8 C a	3.3 ± 4.6 C a	3.3 ± 3.3 C a	2.7 ± 3.1 CD a
	*Botrytis* spp.	0.1 ± 0.3 C b	0.0 ± 0.0 C b	0.8 ± 1.2 C a	0.0 ± 0.0 D b
2020	*Fusarium* spp.	28.1 ± 10.1 C a	0.8 ± 1.0 C c	17.3 ± 6.0 C b	0.4 ± 0.5 B c
*n* = 800 *	*Alternaria* spp.	35.3 ± 6.2 BC b	9.8 ± 3.5 C c	52.1 ± 12.4 A a	5.0 ± 4.1 B c
	*Aspergillus* spp.	1.0 ± 1.2 D a	0.1 ± 0.4 C a	0.3 ± 0.7 E a	0.1 ± 0.4 B a
	*Penicillium* spp.	43.6 ± 18.3 B a	32.0 ± 33.2 B ab	11.8 ± 9.2 CD bc	1.9 ± 3.4 B c
	*Cladosporium* spp.	91.1 ± 4.2 A a	86.8 ± 13.6 A a	30.6 ± 10.2 B b	22.5 ± 19.5 A b
	*Mucor* spp.	0.8 ± 1.4 D a	0.1 ± 0.4 C a	0.5 ± 0.8 E a	0.0 ± 0.0 B a
	*Botrytis* spp.	7.8 ± 3.3 D a	0.6 ± 0.9 C b	3.6 ± 4.2 DE b	0.5 ± 0.8 B b

Note. * n—number of tested seeds in each subgroup, ± standard deviation. In each year, means within each row followed by the same lowercase letters are not significantly different at *p* ≤ 0.01. In each year, means within each column followed by the same uppercase letters are not significantly different at *p* ≤ 0.01.

## Data Availability

Not applicable.
